# Bacteriophage Mu contamination impacts interbacterial competition

**DOI:** 10.1128/iai.00268-25

**Published:** 2025-10-17

**Authors:** Kat Pick, Valeria Tsviklist, Lauren Stadel, Tracy Raivio

**Affiliations:** 1 Department of Biological Sciences, University of Alberta 3158, Edmonton, Alberta, Canada; University of California Davis, Davis, California, USA

## Abstract

Here, we report the identification of bacteriophage Mu contamination in a commonly used *Citrobacter rodentium* DBS100 ∆*cpxRA* mutant strain. After re-constructing a new Mu-free ∆*cpxRA* strain, we independently replicated the results of a recent study by A. Gilliland, C. Gavino, S. Gruenheid, and T. Raivio (Infect Immun 90:e00314-22, 2022, https://doi.org/10.1128/iai.00314-22). The only result from Gilliland et al. that was impacted by the presence of Mu was the outcome of interbacterial competition assays with the ∆*cpxRA* strain, as strains carrying Mu consistently outcompeted susceptible Mu-free competitors. These results are important for the field, as the contaminated DBS100 ∆*cpxRA* mutant strain has been used in six different studies. We believe that the Mu contamination occurred during the construction of the ∆*cpxRA* allele, during the conjugation of DBS100 with a popular Mu-containing donor strain. Our results highlight the importance of using Mu-free conjugal donor strains and how phage contamination can impact bacterial physiology and experimental results.

## MATTERS ARISING

The Cpx envelope stress response is a conserved two-component system comprised of the sensor histidine kinase CpxA and response regulator CpxR which together monitor and maintain the integrity of the bacterial envelope (1). A recent article by Gilliland et al. reported that *Citrobacter rodentium* DBS100 ∆*cpxRA* mutants have a competitive advantage over WT DBS100 in LB (Fig. 7B) (2). We were puzzled by this result, as *C. rodentium* ∆*cpxRA* mutants have been previously demonstrated to have colonization, virulence, and fitness defects (3–7), and Cpx mutants of related species typically have competitive defects (8, 9). Hypothesizing that some type of off-target mutation may have occurred in the ∆*cpxRA* strain, we performed whole-genome sequencing of the WT and ∆*cpxRA* strains. Illumina sequencing was performed by SeqCenter (Pittsburgh, PA, USA) using the Illumina DNA Prep Kit and IDT 10 bp UDI indices and sequenced on an Illumina Nextseq 2000 producing 2 × 151 bp reads. bcl-convert (v3.9.3) (Illumina) was used to demultiplex, trim adapters, and check quality. Trimmed (10) WT reads were first compared against the NCBI reference genome (accession no. CP038008.1) using breseq (11) to identify any laboratory-specific mutations that may have arisen in our stock. These mutations were then applied to the NCBI reference genome using gdtools APPLY (11) to obtain a complete reference genome for comparison against the ∆*cpxRA* strain. Trimmed (10) ∆*cpxRA* reads were then compared against this new reference genome using breseq (11), resulting in six predicted mutations.

**Fig 1:**
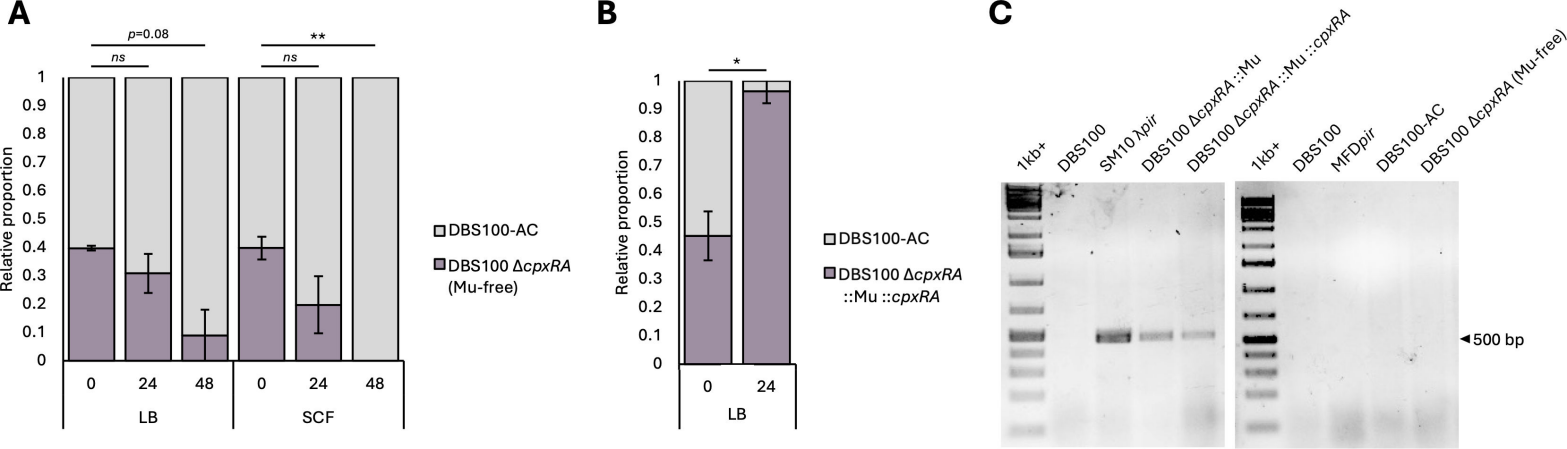
Mu contamination of DBS100 **∆***cpxRA* drives competition outcomes. (**A and B**) Bacterial competition assays were performed as described in reference 1, using the bacterial strains listed in Table 1. Arcsine-transformed values were used to perform paired *t*-tests (*ns P* > 0.05, *P < 0.05, **P < 0.01). (**C**) Colony PCR of strains listed in Table 1, using primers targeting Mu portal protein (CAACCTGCGTGAGGAAGC, GGAATGCCGCCTCATCAATC).

Besides the expected ∆*cpxRA* mutation, we identified two missense mutations (N857D and T828P) in E2R62_02460 (autotransporter outer membrane beta-barrel domain-containing protein) and three SNPs in the intergenic region between two pseudogenes (E2R62_20730 and E2R62_20735). While certain autotransporters have been implicated in *C. rodentium* virulence (12), we did not find any clear links to interbacterial competition in the literature and doubted whether the predicted mutations would confer as strong a competitive advantage as seen by Gilliland et al., leading us to dig deeper into our sequencing results. When reads do not align to the reference genome, breseq outputs them into an unmatched.fastq file. *De novo* assembly of these unmapped reads using SPAdes (13) revealed a 33 kbp contig not present in WT DBS100. BLASTn (14) searches of this contig identified it as bacteriophage Mu (accession no. AF083977.1). Several prophages have been shown to provide a competitive advantage to their hosts (15, 16), leading us to hypothesize that the presence of Mu in the DBS100 ∆*cpxRA* strain is responsible for the previously reported competitive advantage (2). We constructed a new Mu-free DBS100 ∆*cpxRA* strain as described in Gilliland et al., using the Mu-free conjugal donor strain MFD*pir* (17), and repeated the competition assay from Gilliland et al. The Mu-free DBS100 ∆*cpxRA* strain now shows a fitness defect in LB (Fig. 1A), indicating that Mu likely provided a competitive advantage to the original ∆*cpxRA* strain. Interestingly, however, Gilliland et al. found that the ∆*cpxRA* strain still had a competitive defect in simulated colonic fluid (SCF), suggesting that the fitness benefit afforded by Mu is not significant enough to overcome the stressors associated with growth in SCF and was only relevant during competition in LB.

**TABLE 1:** Bacterial strains used in this study

Name	Genotype	Source	Notes
DBS100	*Citrobacter rodentium* ATCC 51459	(18)	
SM10 λ*pir*	*thi-1 thr leu tonA lacY supE recA::*RP4-2-Tc^r^::Mu Km^r^ λ*pir*	(19, 20)	
∆*cpxRA*	DBS100 ∆*cpxRA*	(3)	::Mu
∆*cpxRA* ::*cpxRA*	DBS100 ∆*cpxRA attTn7*::*cpxRA*	(3)	::Mu
MFD*pir*	MG1655 RP4-2-Tc::[∆Mu1::*aac (3)IV*-∆*aphA*-∆*nic35*-∆Mu2::*zeo*] ∆*dapA*::(*erm-pir*) ∆*recA*	(17)	
VT298	DBS100 ∆*cpxRA*	This study	Mu-free
DBS100-AC	DBS100 *xylE::tetRA-amCyan*	(2)	
AG90	DBS100 (pNLP10 *ler*)	(2)	
VT359	VT298 (pNLP10 *ler*)	This study	
AG31	DBS100 (pNLP10 *yebE*)	(2)	
VT360	VT298 (pNLP10 *yebE*)	This study	
AG32	DBS100 (pNLP10 *ygiB*)	(2)	
VT361	VT298 (pNLP10 *ygiB*)	This study	
AG38	DBS100 (pNLP10 *bssR*)	(2)	
VT362	VT298 (pNLP10 *bssR*)	This study	
AG61	DBS100 (pNLP10 *htpX*)	(2)	
VT363	VT298 (pNLP10 *htpX*)	This study	

Tracing the construction of the original ∆*cpxRA* strain (3), it is likely that it was contaminated with Mu during conjugation with χ7213, which has been reported to occur with other Mu-containing conjugal donors such as SM10 λ*pir* (17). If this is the case, then the ∆*cpxRA ::cpxRA* strain that was constructed in the same manner (3) should also contain Mu and be unable to complement the competitive phenotype of the original ∆*cpxRA* strain. The ∆*cpxRA ::cpxRA* strain strongly outcompetes WT DBS100 (Fig. 1B) and was shown by PCR to be contaminated with Mu (Fig. 1C), confirming that the competitive advantage previously observed by Gilliland et al. is independent of the Cpx response and is Mu-driven. Strains constructed via conjugation with the Mu-free donor strain MFD*pir* are Mu-free (Fig. 1C).

**Fig 2:**
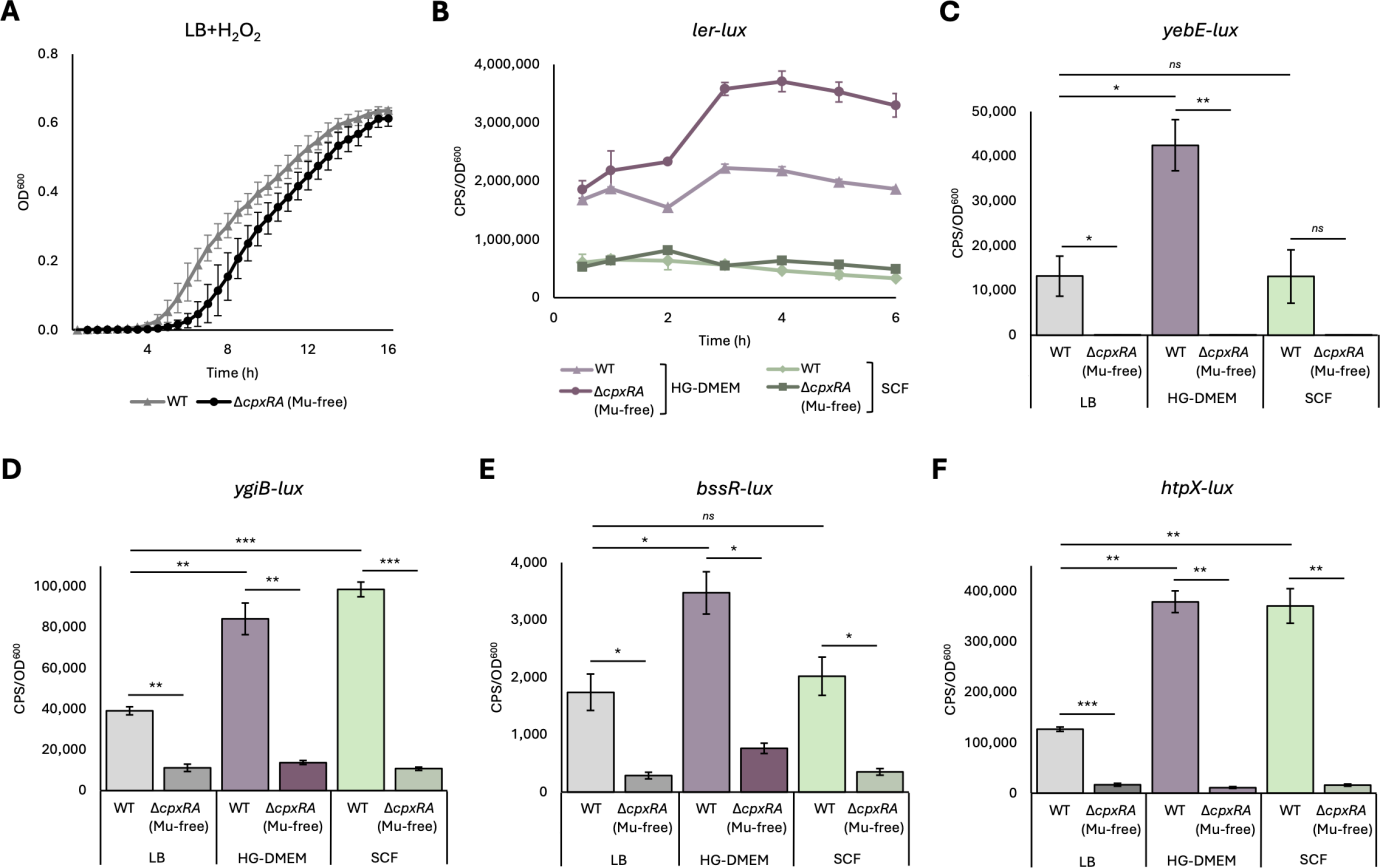
Replication of results from Gilliland et al. with Mu-free DBS100 **∆***cpxRA.* Growth curves (**A**), and luminescent reporter assays (**B through F**) were performed as described in reference 2, using the bacterial strains listed in Table 1. Paired *t*-tests were used to determine statistical significance (*ns P* > 0.05, *P < 0.05, **P < 0.01).

Assembly of the DBS100 ∆*cpxRA* ::Mu strain using SPAdes (13) revealed that Mu is integrated into the *ptrA* pitrilysin gene (locus tag E2R62_10930) between nucleotides 67 and 68. *ptrA* expression was previously shown to be strongly reduced in DBS100 ∆*cpxRA* mutants, leading the authors to hypothesize that *ptrA* may be positively regulated by the Cpx response (6). However, our results suggest that the disruption by *ptrA* by Mu may instead be responsible. In DBS100, *ptrA* is the operon leader of two downstream genes involved in DNA repair, *recB* and *recD*, whose expression is likely also impacted by the disruption of *ptrA* by Mu. To confirm whether any other results from Gilliland et al. were impacted by the presence of Mu, we repeated the experiments presented in Fig. 2, 3A through D, and 5A with our Mu-free ∆*cpxRA* strain. ∆*cpxRA* phenotypes presented in Fig. 1A, 4, 5B of Gilliland et al. were already verified by complementation in the original article (Fig. S5), and ∆*cpxRA* phenotypes presented in Fig. 8 were previously verified by complementation elsewhere (3). We successfully replicated the results from Gilliland et al. Fig. 2, 3A through D, and 5A with the new Mu-free ∆*cpxRA* strains (Fig. 2), indicating that the only phenotype impacted by Mu was the competition assay in LB reported in Fig. 7B of Gilliland et al. We did, however, note that *yebE-lux* and *bssR-lux* expression in WT DBS100 were not significantly increased in SCF compared to LB (Fig. 2C and E), as was observed by Gilliland et al. (Fig. 3A, C). Since we used the strains originally constructed by Gilliland et al. for this experiment, this discrepancy is likely explained by minor differences in media composition. Since SCF is prepared by weighing eight different powders, and a fresh batch is prepared for each experiment, there is likely significant batch-to-batch variability.

In summary, we have identified bacteriophage Mu as a contaminant found in *C. rodentium* DBS100 ∆*cpxRA* and demonstrated that the presence of Mu can impact bacterial competition outcomes. Mu was likely transferred to DBS100 from the conjugal donor strain χ7213 (3), and we strongly urge readers to instead use the Mu-free conjugal donor strain MFD*pir* (17). Strains constructed by conjugation with S17-1 λ*pir*, SM10 λ*pir,* or any of their Mu-containing derivatives should be carefully re-examined, as transfer of Mu from these strains into susceptible recipient strains is known to occur (17). Only one ∆*cpxRA* phenotype reported by Gilliland et al. was impacted by the presence of Mu and does not significantly change the overall conclusions of their study.
